# Performance evaluation of a deep learning model for automatic detection and localization of idiopathic osteosclerosis on dental panoramic radiographs

**DOI:** 10.1038/s41598-024-55109-2

**Published:** 2024-02-23

**Authors:** Melek Tassoker, Muhammet Üsame Öziç, Fatma Yuce

**Affiliations:** 1 Faculty of Dentistry, Department of Oral and Maxillofacial Radiology, Necmettin Erbakan University, Bağlarbaşı Street, 42090 Meram, Konya Turkey; 2 Faculty of Technology, Department of Biomedical Engineering, Pamukkale University, Denizli, Turkey; 3 Faculty of Dentistry, Department of Oral and Maxillofacial Radiology, Istanbul Okan University, Istanbul, Turkey

**Keywords:** Deep learning, Dense bone island, Idiopathic osteosclerosis, Panoramic radiography, YOLOv5, Medical research, Engineering

## Abstract

Idiopathic osteosclerosis (IO) are focal radiopacities of unknown etiology observed in the jaws. These radiopacities are incidentally detected on dental panoramic radiographs taken for other reasons. In this study, we investigated the performance of a deep learning model in detecting IO using a small dataset of dental panoramic radiographs with varying contrasts and features. Two radiologists collected 175 IO-diagnosed dental panoramic radiographs from the dental school database. The dataset size is limited due to the rarity of IO, with its incidence in the Turkish population reported as 2.7% in studies. To overcome this limitation, data augmentation was performed by horizontally flipping the images, resulting in an augmented dataset of 350 panoramic radiographs. The images were annotated by two radiologists and divided into approximately 70% for training (245 radiographs), 15% for validation (53 radiographs), and 15% for testing (52 radiographs). The study employing the YOLOv5 deep learning model evaluated the results using precision, recall, F1-score, mAP (mean Average Precision), and average inference time score metrics. The training and testing processes were conducted on the Google Colab Pro virtual machine. The test process's performance criteria were obtained with a precision value of 0.981, a recall value of 0.929, an F1-score value of 0.954, and an average inference time of 25.4 ms. Although radiographs diagnosed with IO have a small dataset and exhibit different contrasts and features, it has been observed that the deep learning model provides high detection speed, accuracy, and localization results. The automatic identification of IO lesions using artificial intelligence algorithms, with high success rates, can contribute to the clinical workflow of dentists by preventing unnecessary biopsy procedure.

## Introduction

Idiopathic osteosclerosis (IO) of the jaws are localized, non-expansive radiopacities of unknown cause^1^. Other known names are bone scarring, dense bone island, enostosis, and focal periapical osteopetrosis. They are usually seen in the mandible and premolar-molar regions. IOs are non-encapsulated lesions with homogeneous internal structures, smooth outer borders, and no radiolucent margins^2^. Their size can vary from 2–3 mm to 1–2 cm. They can be seen in the roots of the teeth, between the roots, or in any area on the jaw arch independent of the teeth^3^. Rarely, they may cause external root resorption in cases associated with the tooth's root, but this is self-limiting, and the involved tooth is vital^4^. Apart from the jaws, they can also be seen in the pelvis, femur, and other long bones^5^. IO consists of mature vital bone, which histologically does not contain medullary spaces and does not contain inflammatory infiltrate^6^. In the literature, studies conducted with panoramic, periapical radiographs and cone-beam computed tomography have reported a frequency of 0.10%^6^ to 31%^7^. Since IO lesions are asymptomatic, they are diagnosed incidentally on radiographs taken for different reasons^4^. These lesions developing in the cancellous bone do not require treatment. Clinical follow-up is sufficient^8^. However, they must be differentiated from radiopacities that require treatment due to inflammatory or systemic disease^2^.

The term "artificial intelligence (AI)" is used when a machine can carry out tasks typically associated with human minds, like "learning" and "problem-solving." Learning is a crucial element for machines, making machine learning a subset of AI. In recent years, substantial efforts have been dedicated to advancing machine learning, raising expectations for the capabilities of machines. Deep learning represents a step in this progression. Founded on artificial neural networks, deep learning is a subset of machine learning. These networks strive to replicate both the structure and functionality of the human brain, enabling them to autonomously acquire knowledge and make intelligent decisions without the need for explicit programming. In numerous applications, deep learning models exhibit superior performance compared to both shallow machine learning models and conventional approaches to data analysis^9,10^. In image analyses using conventional machine learning methods, it is necessary to perform feature extraction and feature selection processes initially. Since there are numerous methods for these tasks, researchers repeatedly conduct experiments to find the most suitable procedure. Deep learning models, on the other hand, are highly advantageous in terms of cost compared to conventional machine learning methods because they autonomously extract and select features across layers. They can automatically learn and capture relevant features throughout the training process, eliminating the manual feature engineering procedures needed in conventional AI methods^11,12^. Deep learning models generally encounter tasks such as segmentation, classification, pose estimation, and object detection in computer vision. For the widely used object detection task in computer vision, two main approaches are suggested: single-shot (e.g., Single Shot Multibox Detector, You Only Look Once) and two-shot (e.g., Region-based Convolutional Neural Network, Fast Region-based Convolutional Neural Network, Faster Region-based Convolutional Neural Network, Region-based Fully Convolutional Networks, Mask Region-based Convolutional Neural Network). Two-shot approaches involve using a proposal network in the first stage to identify potential object locations on the image, followed by classifying and predicting the position of the identified proposal regions in the second stage. On the other hand, single-shot object detection approaches process the image in a single pass to perform both object localization and classification. Single-shot models offer advantages such as fast detection, lower parameter computation cost, reduced power consumption on resource-constrained devices, real-time performance with quick detection and low inference time, simple model structures, lower labeled image requirements, and efficient memory usage on constrained devices. You Only Look Once (YOLO), a Convolutional Neural Network (CNN)-based single-shot detector, analyzes a small number of labeled images in a single pass, showcasing many of the aforementioned advantages. Numerous studies have indicated its superiority in terms of both speed and accuracy over competitors performing the same object detection task. The YOLO family has been successfully applied in various fields, including both industrial and medical domains^13–17^.

As a result of the recommendation of deep learning algorithms to the literature, detection^18^ and classification^19^ related to the diagnosis of diseases and segmentation of organs^20^ can be performed using different medical images. These improvements can be overcome by the low inter-observer reliability^21^. In recent years, deep learning research has gained momentum in oral radiology^22^. Age estimation^23^, diagnosis of caries^24^, evaluation of periodontal bone loss^25^, mental foramen^26^ and mandibular canal segmentation^27^, diagnoses of jaw cysts and tumors^28^, detection of pulpal calcification^29^, detection of maxillary sinus lesions^30^ and classification of osteoporosis^31^ are some of the studies in this field. In computer-aided diagnosis (CAD) studies, imaging methods such as periapical, panoramic, cephalometric, and cone-beam computed tomography are used to detect and classify diseases^32^. Panoramic radiographs help examine large areas of the jaws^3^. However, panoramic radiographs inherently demonstrate complexity. These radiographs may include artifacts, indistinct boundaries within bony structures, irregular shapes and borders of the mental foramen, as well as jawbones characterized by irregular curvatures and density variations. Furthermore, irregularities in tooth alignment or missing tooth fragments, the presence of implants and fillings, digital noise, and fluctuations in contrast levels are commonly observed features in panoramic radiographs^33^. Therefore, the developed CAD systems need to work adaptively with all complex panoramic radiographs. Since IO shows a hyperdense and radiopaque appearance, it is essential to distinguish it from other radiopaque lesions that can be viewed on different panoramic images. Knowing the imaging characteristics of IO on panoramic radiographs is stated to prevent unnecessary biopsy procedures^6^. Considering the highlighted advantages and disadvantages of panoramic radiographs, automatic detection of IO on panoramic images using an artificial intelligence-based deep learning model could help dentists as a computer-assisted clinical decision support system. Exceptionally few studies perform automatic IO detection with deep learning models^34^. This study investigated the automatic detection and localization success of IO on panoramic radiographs with different features and a small dataset, utilizing the single-shot YOLOv5 algorithm, a popular model within the YOLO family, which possesses the advantages mentioned above. The hypothesis of this study was that the model's ability to identify focal radiopacities of unknown etiology in the jaws would showcase its potential as a valuable tool for incidental detection during routine dental panoramic radiographic examinations.

## Materıals and methods

### The study design and sampling

This study was carried out retrospectively through panoramic radiographs taken for various diagnostic reasons at the Faculty of Dentistry, Department of Oral and Maxillofacial Radiology. The study protocol was approved by Necmettin Erbakan University, Faculty of Dentistry, Ethics Committee for Non-Drug and Medical Device Research (No:17-127) and conducted in accordance with the principles defined in the Declaration of Helsinki, including all revisions. Informed consent is not required for retrospective studies by the Necmettin Erbakan University, Faculty of Dentistry, Ethics Committee for Non-Drug and Medical Device Research. The current standards for diagnosing IO included the following characteristics: a clearly outlined radiopaque area exceeding 3mm in size associated with normal bone, exhibiting a round, elliptical, or variably irregular shape, and lacking a surrounding radiolucent rim^35,36^. Based on these criteria, the study included panoramic radiographs from 175 patients of both genders aged 18 and older, all exhibiting at least one radiopaque lesion consistent with the definition of idiopathic osteosclerosis.

The exclusion criteria included lesions exhibiting the following characteristics:

- Connection with persistent inflammation characterized as condensing osteitis.
- Lesions displaying a combination of radiopaque and radiolucent features, such as diffuse sclerosing osteomyelitis.
- Hypercementosis, cementoblastoma, odontoma, non-malignant fibro-osseous conditions like periapical cemento-osseous dysplasia and focal cemento-osseous dysplasia.
- Discernible remnants of deciduous or permanent teeth.
- Individuals diagnosed with Gardner's syndrome, familial polyposis of the colon, and other conditions involving bone metabolic disturbances (as per patient file information).
- Images with insufficient image quality and artifacts that prevent the diagnosis of IO.

### Image acquisition and radiographic evaluation

All panoramic radiographs reviewed were taken by a single device and acquired with a 2D Veraviewpocs (J MORITA MFG corp, Kyoto, Japan) digital panoramic X-ray device with 70 kVp, 5 mA, and 15 s irradiation time in accordance with the exposure protocols determined by the manufacturer. Two oral and maxillofacial radiologists with experience of 11 years (MT) and three years (FY) conducted the examinations through i-Dixel (J Morita MFG Corp., Kyoto, Japan) software. Inter- and intra-observer agreements were evaluated using Cohen’s Kappa statistics and showed excellent consistency in defining the IO (0.81–0.99). Localized well-defined radiopacities in different sizes associated with normal bone, without radiolucency in the periphery, were defined as IO^35^. All lesions in both the upper and lower jaws were examined.

### Dataset and image pre-processing

**Figure 1:**
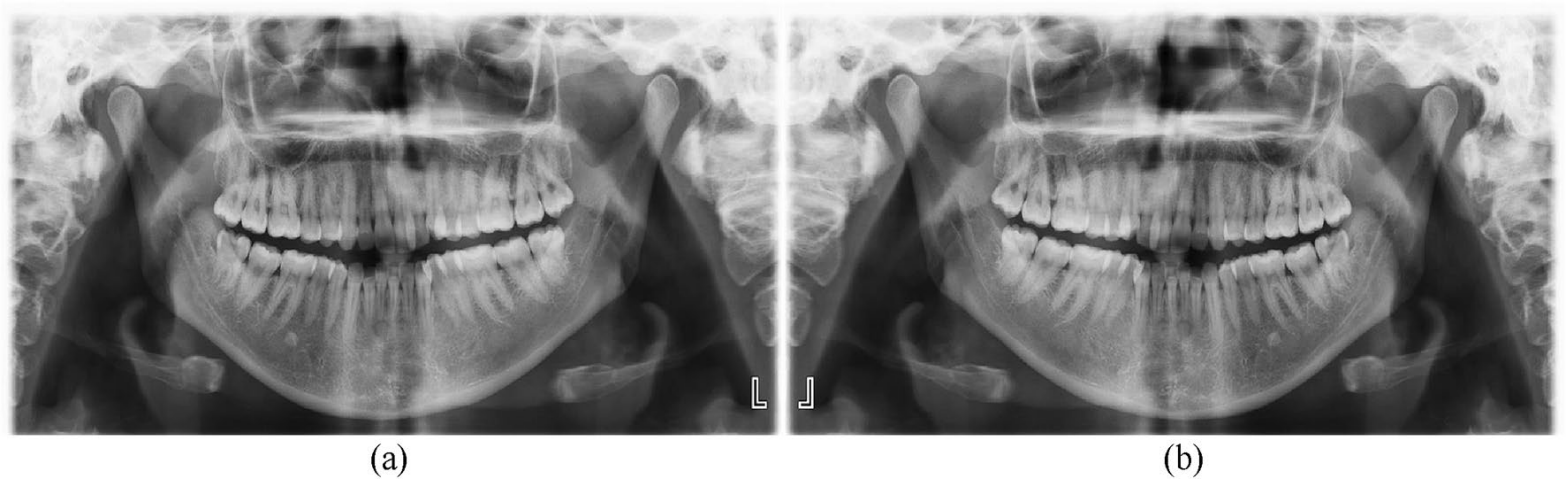
(**a**) Original panoramic radiograph. (**b**) Horizontally flipped panoramic radiograph.

One hundred seventy-five panoramic radiographs diagnosed with IO from the Dental School database. Since panoramic radiographs were in different image extensions such as *.png, *.gif, *.tiff, *.jpg, and *.jpeg, they were all converted to *.jpg format. Due to variations in pixel sizes among panoramic images, a consistent size of 1930 × 1024 was applied to all radiographs through the use of the bilinear interpolation method. IOs show pattern changes close to the mandibular bone areas. For this reason, the dimensions were kept at the maximum level so that the region was not distorted in the resizing process. For data augmentation, all images were flipped horizontally. Thus, the number of data was increased to 350 radiographs. The fillings, implants, tooth loss, different tone levels, and different contrast levels in the radiographs provided richer data. An original and horizontally flipped panoramic radiograph is given in Fig. 1.

### Image annotation

**Figure 2:**
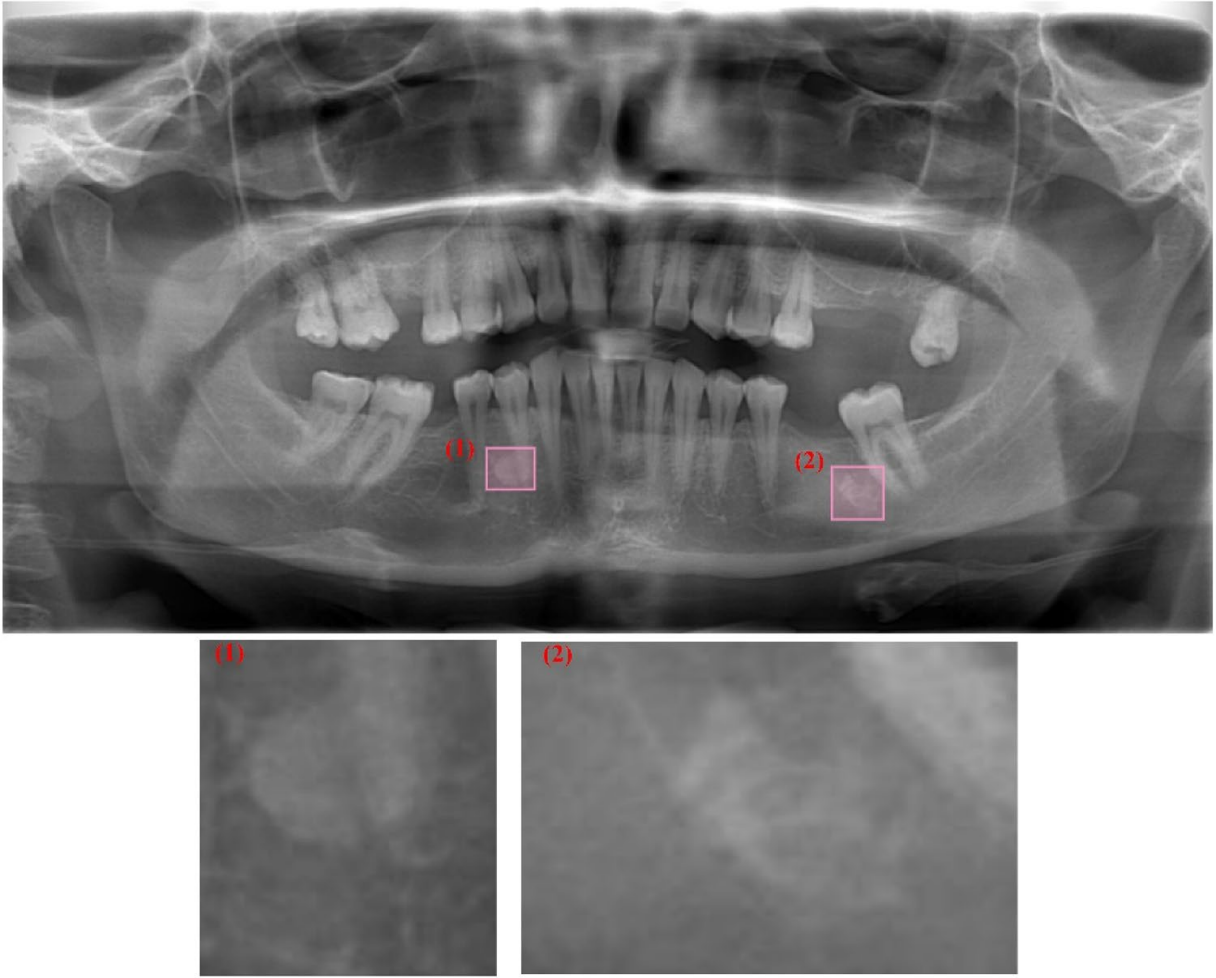
Two annotated IO on high-contrast panoramic radiography and their zooming for close monitoring of pattern changes.

**Figure 3:**
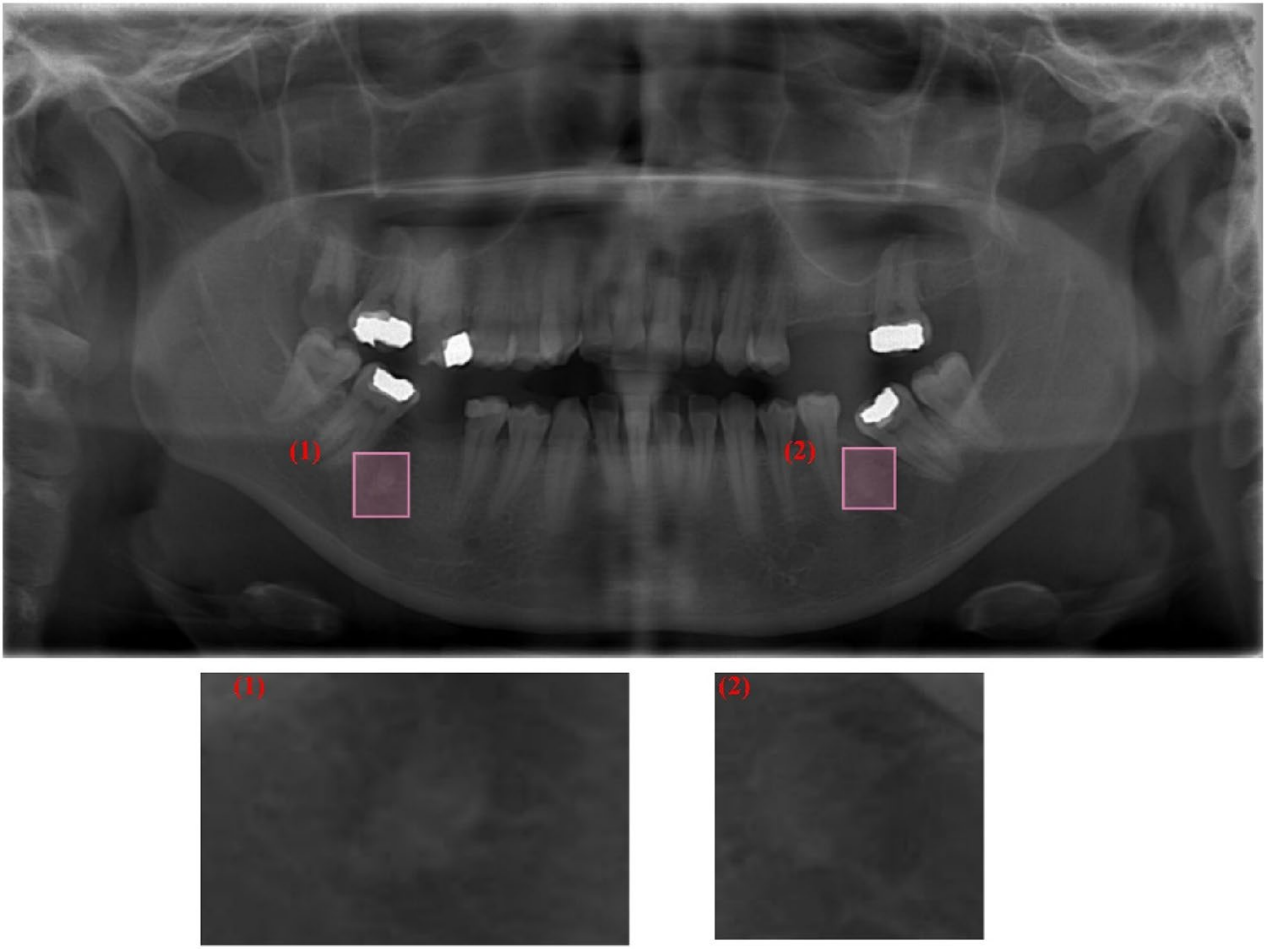
Two annotated IO on low-contrast panoramic radiography and their zooming for close monitoring of pattern changes.

The bounding boxes of IO in the resized and augmented images were annotated using the free browser-based *makesense* application (https://www.makesense.ai/). Since IOs are a condition that causes excessive hardening of bones, increases in bone density are observed in the areas where lesions are seen on panoramic radiography (Figs. 2, 3). Therefore, radiologists have conducted the annotation process by considering criteria such as density, consistency, location, and exclusion of other lesion types in cases where an increase in bone density is observed. As seen in Figs. 2 and 3, radiographs with different contrasts and patterns have been carefully annotated by drawing bounding boxes, as they tend to exhibit color tones closely resembling the pattern of the mandibular lesion region. After the annotating process, the label information was downloaded as a separate *.txt file for each image in YOLO format. These files contain the bounding box coordinates of the IOs. Figures 2 and 3 show the bounding boxes of the lesion regions and the pattern changes at close range in high and low-contrast panoramic radiographs, respectively.

### YOLOv5 deep learning algorithm

YOLO is a powerful and fast real-time object detection method based on a regression method using CNN. This architecture transforms the problem into a regression solution to obtain the location and classification information of the object according to the loss function^37,38^. YOLOv2^39^, YOLOv3^40^, YOLOv4^41^, and YOLOv5^42^ versions of YOLO algorithms have been presented to the literature since 2016. Shortly after YOLOv4 was introduced to the literature in April 2020^41^, YOLOv5 was released by Glen et al.^42^. YOLOv4 generates high-size weight files after training using the high-performance DarkNET framework. YOLOv5 offers easier usage compared to DarkNET in the Pytorch environment, and the weight file of the model has been reduced by approximately 90% compared to YOLOv4. The YOLOv5 algorithm has different architectures as nano (n), small (s), medium (m), large (l), and Xlarge (x) according to model and parameter size (https://github.com/ultralytics/yolov5). In this study, the m version of the YOLOv5 algorithm, the medium model in terms of the number of parameters and network size, was used to detect IO^42^. The YOLOv5 architecture consists of four main components: Input: pre-processing component, Backbone: CSPDarknet component, Neck: PANet component, and Head: Prediction or YOLO Layer component. Different methods such as mosaic data augmentation, auto-learning bounding box, the cross-stage partial network, pooling, convolutions, spatial pyramid network, and concatenate function have various tasks in components in the architecture. The components that create the architecture are explained below respectively^43–45^.

#### Input (first component)

The input terminal performs pre-processing on the image data, such as adaptive anchor, mosaic data augmentation, and adaptive image scaling. YOLOv5 integrates the adaptive anchor frame calculation module, which allows the initial anchor frame size to be adjusted automatically when the incoming dataset changes. Images used in experiments can have different row and column sizes. Input images are automatically scaled to speed up training and inference times and reduce training parameters.

#### Backbone (second component)

The pre-processed images serve as input to the Backbone module, which is the second element responsible for extracting features in the architecture. The DarkNET-based backbone module uses a cross-stage partial network (CSP)^46^ and spatial pyramid pooling (SPP)^47^ to extract feature maps of different sizes from the input image. Multiple convolutions and pooling operations are performed to obtain the feature maps. The SSP structure extracts features from the same feature map at different scales and performs procedures to improve detection accuracy. The BottleneckCSP structure reduces the computational load and increases the inference speed in the detection process.

#### Neck (third component)

Within the neck module, the integration of PANet (Pixel Aggregation Network)^48^ and FPN (Feature Pyramid Network)^49^ is implemented. PANet is structured upon the FPN topology, facilitating the transfer of features rich in semantic information from the top to the bottom. Thus, the PANet structure can localize high-power feature maps, which take precedence over low-feature maps. PANet and FPN structures are tightly interconnected. Backbone fusion leverages features from different network layers to the Neck module to further enhance sensing capability. These approaches strengthen the location accuracy of the target object.

#### Head (fourth component)

**Figure 4:**
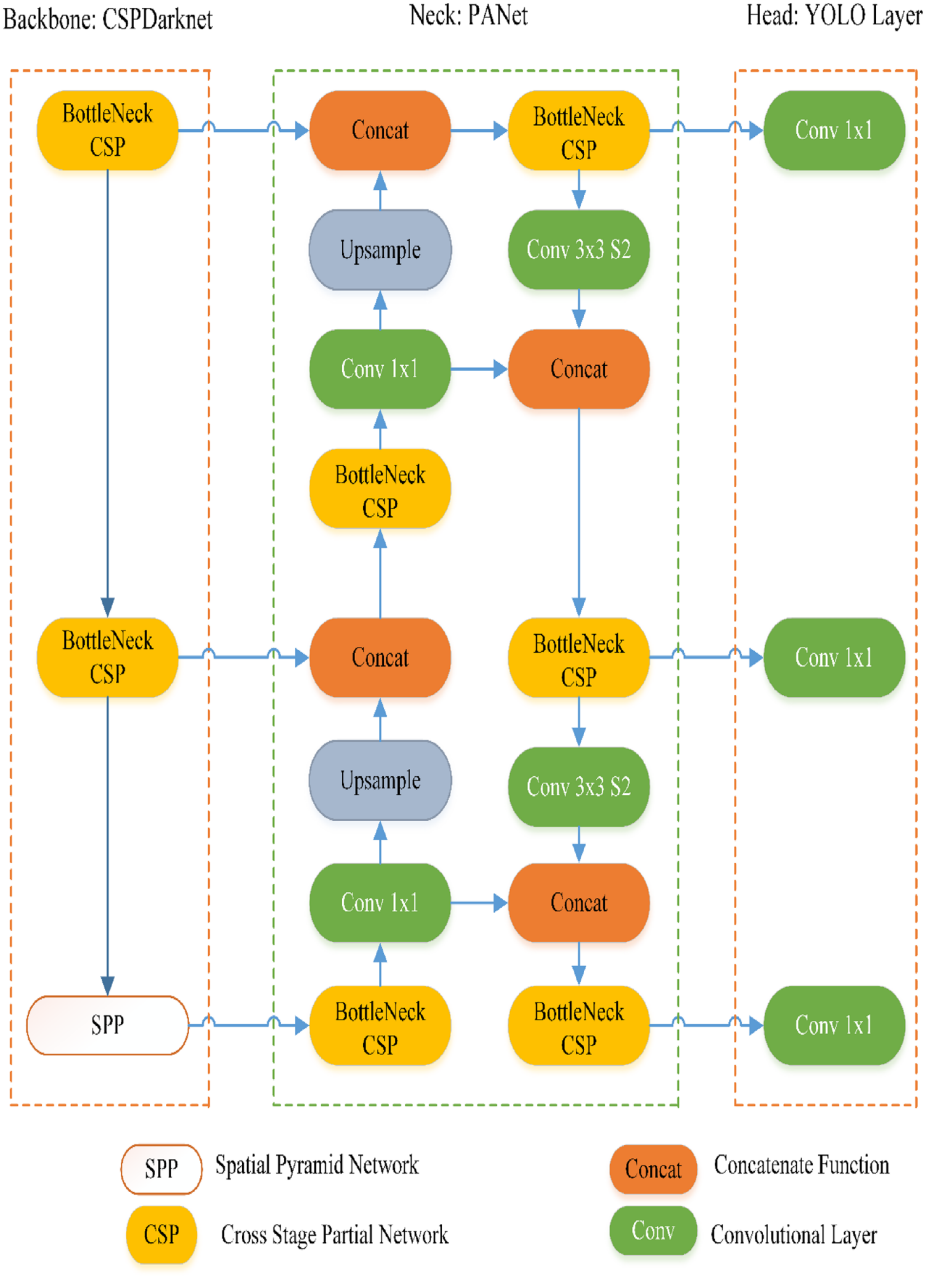
The YOLOv5 architecture^45^.

The Head module, alternatively known as the YOLO Layer or Prediction Layer, represents the final component of the architecture. Within the YOLO Layer module, the operations necessary for object recognition output are executed, encompassing parameters such as size, score, class, location, and bounding box information. The head output estimates target objects of different sizes from the feature maps obtained in the previous step. A schematic representation of the YOLOv5 architecture is given in Fig. 4.

**Figure 5:**
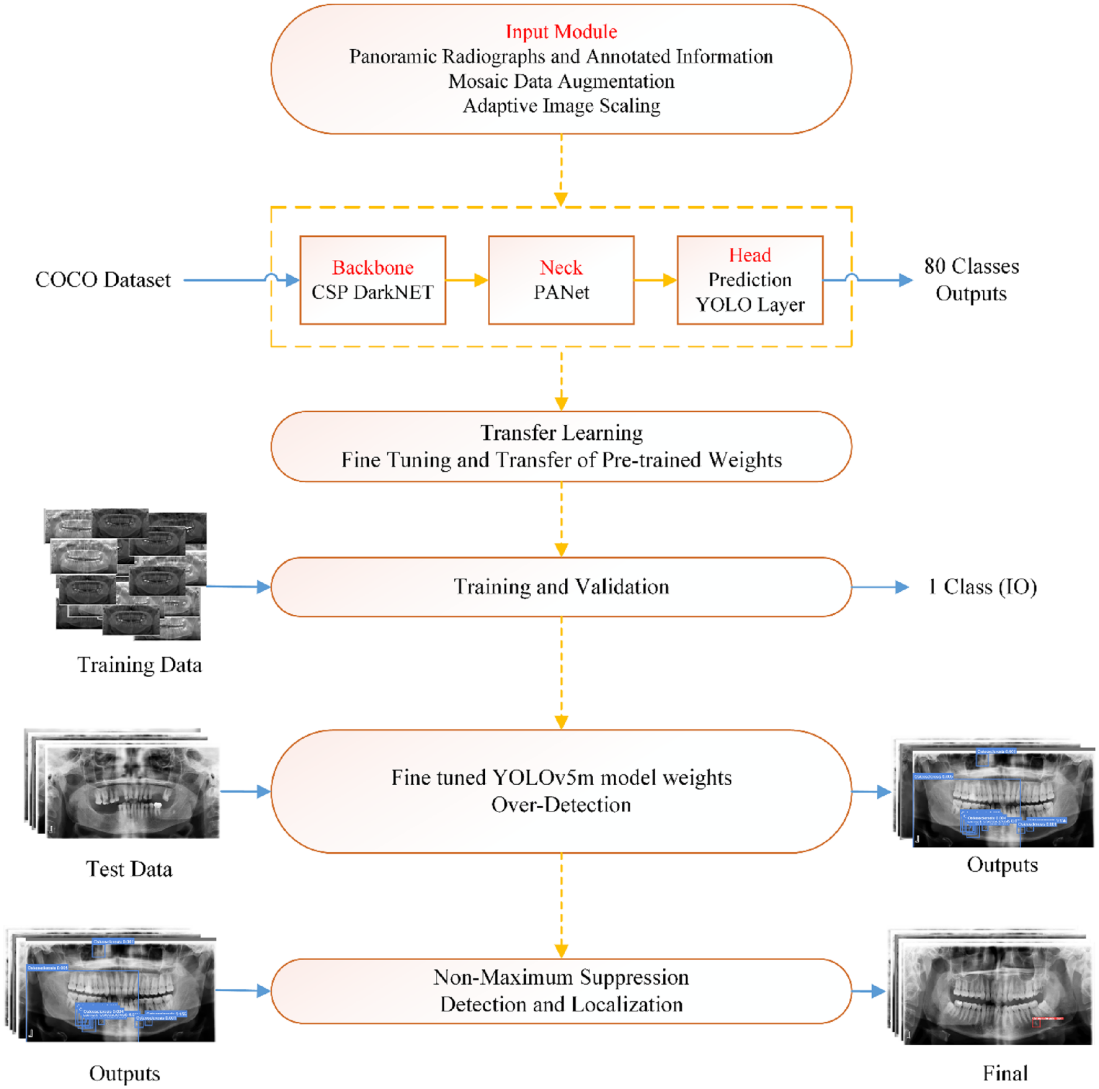
The process from the entry of annotated panoramic radiographs into the YOLOv5 model until the application of the last-stage non-maximum suppression.

For this study, we downloaded the YOLOv5 library from the GitHub repository published by Glen et al.^42^. This library has pre-trained models with the COCO dataset, which outputs eighty classes. To use pre-trained weights in this study, fine-tuning and transfer learning processes were performed. The model outputs were set for IO detection only and loaded into the library. The YOLOv5 algorithm locates a bounding box by detecting many candidate regions where it detects objects as a result of training and testing. Then, the non-maximum suppression technique was applied to prevent the over-detection problem to ensure that the bounding box with the highest confidence score remained on the image. Figure 5 shows the process from the entry of annotated panoramic radiographs into the YOLOv5 model until the application of the last-stage non-maximum suppression.

### Model training and testing

The YOLOv5m model was trained and tested with a Linux-based cloud computer provided by the Pro version of the Google COLAB product (TESLA P100, 32GB RAM). The model parameters were determined as epoch number 600, mini-batch size 4, and optimization method Stochastic Gradient Descent (SGD). Other parameters were left as default. The dataset was randomly divided into 245 training (≌ 70%), 53 validation (≌ 15%), and 52 test (≌ 15%). As a result of the training process, the system gave a weight file named “last.pt”. Then, 52 panoramic images the system had not seen before were tested using the “last.pt” weight file, bounding boxes were drawn to the coordinates of the IO-detected on the images, and confidence scores were written. By applying the non-maximum suppression technique, bounding boxes under a confidence score of 0.25 were suppressed.

### Performance criteria

**Figure 6:**
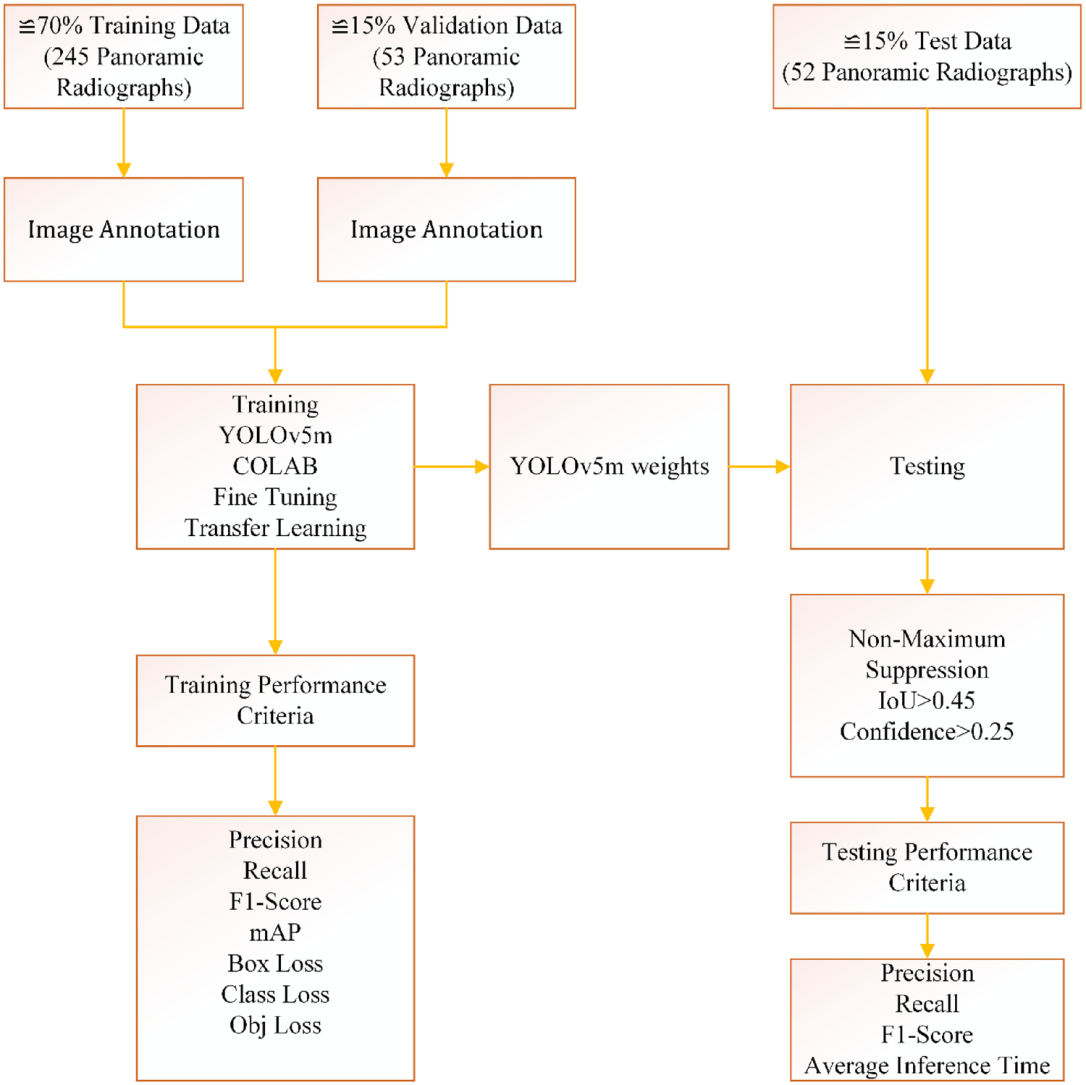
The flow chart of dividing the data, training, and testing processes.

The performance criteria of the model were evaluated in two stages: training and testing. The Weights & Biases (https://wandb.ai/site) platform was integrated with COLAB, and the training process was monitored in real time. The performance of the training process was examined with precision, recall, F1-score, mean Average Precision (mAP) values, loss values, and graphical representations. Loss values are a performance criterion obtained due to minimizing the loss function in the training process. If these values are close to zero, the training is successful. The Average Precision (AP) is defined as the area under the precision-recall curve. The mean Average Precision score is expressed by calculating the mean AP over all classes [Eq. (1)]. High AP and mAP values show that the training with the deep learning algorithm is so successful [Eq. (2), k: number of classes]. Training performance criteria were obtained automatically by running the YOLOv5 algorithm on the COLAB platform. Fifty-two test images were passed from trained model weights, taking into account the parameters of confidence score > 0.25 and IoU > 0.45 (default values of YOLOv5), and bounding boxes determined by the non-maximum suppression were drawn on the images. During this process, the average inference time was calculated. Two radiologists evaluated these test images, and the results were scored as True Positive (Radiologists detect there is, the algorithm detects there is), False Positive (Radiologists detect there is no, the algorithm detects there is), False Negative (Radiologists see there is but the system cannot find). Precision [Eq. (3)], recall [Eq. (4)], and F1-score [Eq. (5)] values were calculated using True Positive (TP), False Positive (FP), and False Negative (FN). The Precision value shows how well true positives are predicted in all the bounding boxes the algorithm detects. The Recall value, on the other hand, shows how well the true positives are detected for all regions the radiologist saw as IO. Recall and precision are generally inversely proportional to each other. F1-score is the harmonic average of the recall and precision value. Figure 6 shows the flow chart for dividing the data, training, and testing processes.

1 $$AP_{k} = \int\limits_{0}^{1} {P_{k} } (R_{k} )dR_{k}$$

2 $$mAP = \frac{1}{k}\sum\limits_{i = 1}^{k} {AP_{i} }$$

3 $$precision = \frac{TP}{{TP + FP}} = \frac{TP}{{allDetections}}$$

4 $$recall = \frac{TP}{{TP + FN}} = \frac{TP}{{allGroundTruths}}$$

5 $$F1 - score = 2*\frac{Precision*Recall}{{Precision + Recall}}$$

### Ethics approval

This study was conducted at the Faculty of Dentistry, Necmettin Erbakan University, Department of Dentomaxillofacial Radiology, with the approval of the Necmettin Erbakan University, Faculty of Dentistry, Ethics Committee for Non-Drug and Medical Device Research (No. 17/127) and was performed according to the stipulations laid out by the Declaration of Helsinki.

### Informed consent

Not Applicable. Informed consent is not required for retrospective studies by the Necmettin Erbakan University, Faculty of Dentistry, Ethics Committee for Non-Drug and Medical Device Research.

### Conference presentation

This study was presented as an oral presentation at the 26th TDB International Dental Congress, on 8–11 September 2022, Istanbul, TURKEY.

## Results

### Model training and testing results

**Figure 7:**
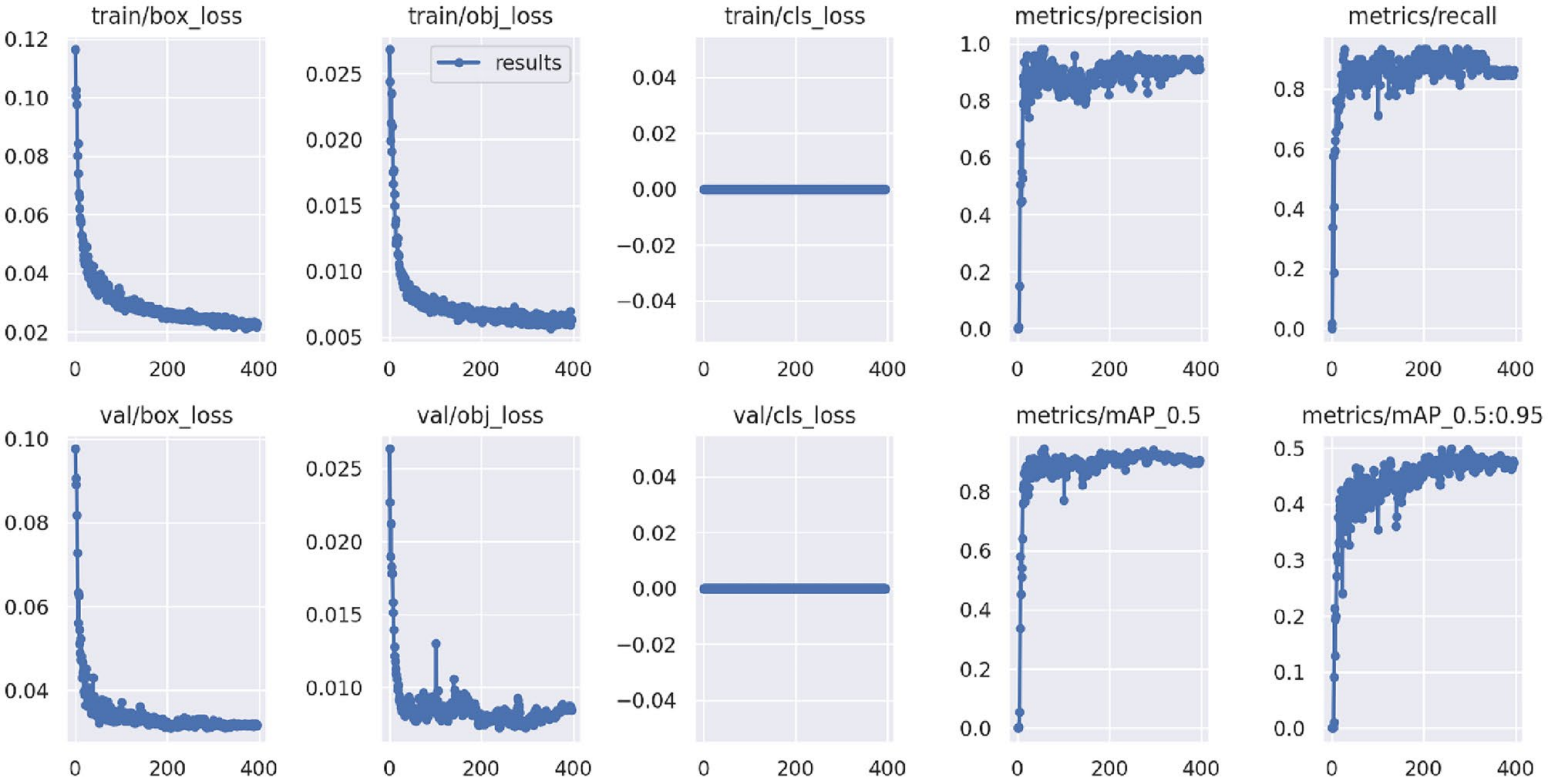
Graphs of different loss and accuracy values according to the epoch progression.

The training process took 1.285 h on the COLAB platform. Parameter input has been set for the network to train for 600 epochs. However, since the system did not significantly improve in the last 100 epochs, it was automatically terminated at the 397th epoch (early stopping). As a result of the training process, in which 20,852,934 parameters were calculated, a weight file of 42.1 Megabytes was obtained. The weights were given the default threshold parameters of the YOLOv5m “detect.py” Python script (IoU > 0.45 and confidence score > 0.25). Out of 56 IOs in 52 test images, 52 IOs were correctly detected. However, one region was detected incorrectly, and 4 IOs could not be detected. The performance criteria results of the training and testing processes are given in Table 1. In Fig. 7, graphs of different loss and accuracy values are given according to epoch progression.

**Table 1:** Results of performance criteria of training and testing processes.

	Training (validation)	Testing
Radiographs	53	52
IO	59	56
Precision	0.902	0.981
Recall	0.932	0.929
F1-score	0.917	0.954
mAP	0.939	–
Box loss	0.023	–
Class loss	0.000	–
Obj loss	0.006	–
Average inference time	–	25.4 ms

### Results of localization and visualization

**Figure 8:**
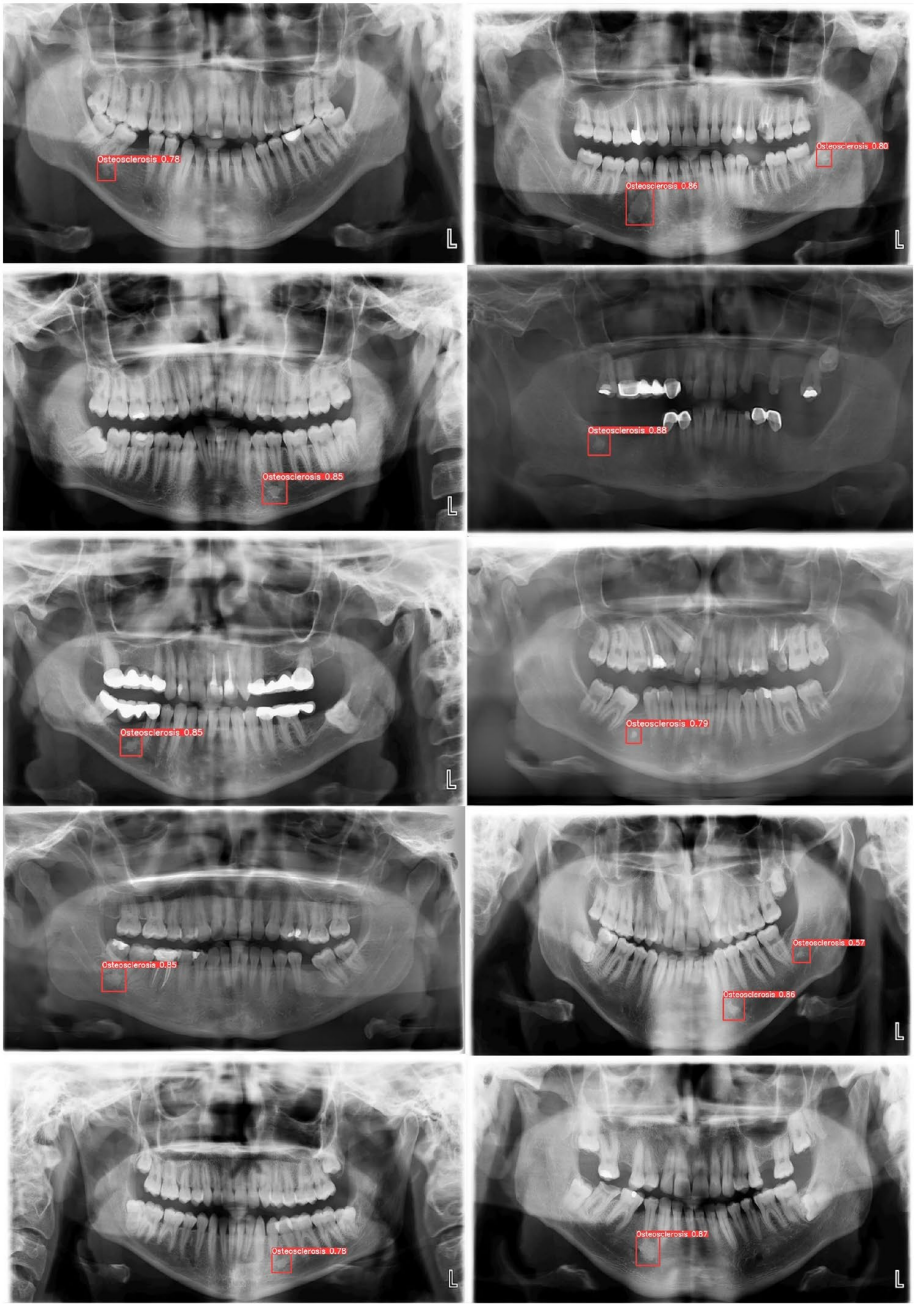
Automatic IO detection and localization of the YOLOv5 deep learning model on ten different panoramic test images with different characteristics.

Visualization and localization were performed using the weights obtained from 52 panoramic test radiographs. In the evaluation made by the radiologists, it was observed that the borders of the IOs on the image could be taken into the bounding box exactly. In each image, IOs have different sizes, environmental irregularities, contrast, and pixel tone levels. At the same time, each dental panoramic radiograph has distinct contrast, pixel tone levels, tooth shapes, and jaw structures. Despite the mentioned negative features of both IOs and panoramic radiographs, the trained model accurately and rapidly detected and localized the vast majority of data it had never seen. Figure 8 shows the automatic IO detection and localization of the YOLOv5 deep learning model on ten panoramic test images with different characteristics.

## Discussion

Artificial Intelligence (AI) applications in radiological images are hot topics frequently studied in the literature recently. Especially, computer-aided decision support systems, capable of automatically analyzing radiological images through deep learning algorithms, assist medical doctors and dentists in their diagnostic processes. These applications can accelerate the diagnosis and treatment processes and increase patient welfare. As different deep learning models are proposed in the literature, there is an increase in segmentation, recognition, classification, and detection studies on radiological tooth images. However, there are still many issues to be studied in the AI-based prediction of many dental diseases and cases. Panoramic radiographs are X-ray-based images, and it is the most widely used medical imaging technique in dental clinics, which provides a panoramic view of many structures. Thus, a single image can analyze structures such as jaw bones, teeth, pathological formations, cysts, tumors, impacted teeth, caries, and fillings. However, panoramic radiographs are inherently very complex due to the different mouth structures of individuals and other differences in medical image acquisition (Figs. 1, 2, 3, 8). Panoramic radiographs may include the following complex formations: artifacts, indistinct boundaries in bone structures, jawbones with irregular curvatures and density levels, irregular shapes and borders of the mental foramen, irregular tooth alignments, missing tooth fragments, implants, fillings, digital noises, and varying levels of contrast^33^. AI-based CAD systems designed for panoramic radiographs are expected to be adaptive decision support systems that can provide successful results in all images, considering the abovementioned complex situations. It has been reported that deep learning-based CAD systems performed on panoramic images give successful results. Tooth segmentation and numbering^50,51^, impacted tooth detection^52^, residual tooth^53^, dental calculus^53^, filling^53^, and caries^54^ are some of the studies presented in the literature. This study proposes an approach that performs automatic detection and localization of IO with the YOLOv5m deep learning algorithm on panoramic radiographs.

IO lesions are non-expansive localized bone radiopacities and do not pose a health risk^6^. IO's internal structure, usually seen as radiopaque masses in the mandible and premolar-molar region, has a homogeneous pattern. Its external structure consists of elliptical, rounded, and irregular borders (Figs. 2, 3, 8). Radiographic evaluation of the morphological features of IO and its relationship with anatomical structures is essential for pre-surgical planning in clinical interventions such as maxillofacial trauma and implants^55^. In addition, knowing the radiological features is very important to prevent unnecessary biopsy procedures for the patient^6^. Detailed radiological evaluations are an essential guide for distinguishing from other radiopaque lesions such as condensing osteitis, cemental dysplasia, ossifying fibroma, osteoma, osteoblastoma, cementoblastoma, and odontoma that may need treatment^56^. In a study performed on 6154 panoramic images, the incidence of IO in the Turkish population was 2.4%^57^. In another study, the incidence of other radiopaque lesions was lower in percentage than IO^58^. Therefore, the clinical collection of many panoramic images with radiopaque masses is a long and laborious process. Routine use of computed tomography for imaging and diagnosis of IO was not recommended^36^. For these reasons, the performance of the deep learning model for the presence and localization of IO of different shapes and sizes in panoramic radiographs, which is relatively more common than other radiopaque masses, was evaluated on a small data set.

In this study, training, validation, and testing processes for automatic detection and localization of IO in panoramic images were carried out using Google's paid COLAB Pro product, which allows the use of a powerful Linux-based virtual computer. In 53 validation images with 59 annotated regions, precision of 0.902, recall of 0.932, F1-score 0.917, mAP 0.939, box loss value 0.023, class loss value 0.0, and obj loss 0.006 were obtained (Table 1). The fact that the performance values were very close to one shows that the IO detection in training was carried out successfully, and the loss values close to zero indicate that the loss function was ideally minimized. When the accuracy and loss graphs were examined according to the epoch number, it was observed that the training process was successful (Fig. 7). Using the YOLOv5 weights obtained by training and then applying the non-maximum technique, IO lesions in 52 test images were automatically identified, as shown in Fig. 8. Of 56 IO regions in 52 test images, 52 were correctly detected. One area was seen incorrectly, and four lesions could not be detected. High success values were obtained with 0.981 precision, 0.929 recall, 0.954 F1-score, and an average inference time of 25.4 ms test performance criteria. Rapid and accurate detection was performed despite the large image size.

Despite the different contrast values in the radiographic images taken from the X-ray device (Figs. 2, 3), there has been high success in detecting IOs. While artifacts, such as the superpositions of the hyoid bone or cervical vertebrae onto the mandibular anterior region, can introduce bias to the algorithm and cannot be eliminated from panoramic images, the algorithm has demonstrated a high level of accuracy in the localization process. The IO lesion exhibited similar pattern features to the mandibular trabecular structure in certain test images (refer to Figs. 2 and 3), yet the success rate remained high. The diagnostic capabilities of deep learning have been extensively studied in the context of identifying radiolucent or mixed radiolucent-radiopaque jaw lesions. However, there is currently a lack of adequate data pertaining to radiopaque lesions such as odontoma, osteoma, and IO. Yang et al. identified 1602 radiolucent jaw lesions on panoramic radiographs using YOLO v2 and classified them as dentigerous cysts, odontogenic keratocysts, and ameloblastoma^59^. The diagnostic accuracy of YOLO v2 resulted in 0.663, the precision value was 0.707, and the recall was 0.680. Kwon et al. classified jaw cysts and tumors as dentigerous cysts, periapical cysts, odontogenic keratocysts, and ameloblastoma with YOLO v3 on 1282 panoramic radiographs and obtained 78.2% sensitivity, 93.9% specificity, 91.3% accuracy, and 0.86 AUC values^32^. In another deep-learning study that used cone-beam computed tomography, the reliability of correctly detecting a radiolucent periapical lesion was found to be 92.8%^60^. Previous studies conducted with YOLO v2^59^ and YOLO v3^32^ have successfully diagnosed radiolucent or mixed radiolucent-radiopaque jaw lesions. The YOLOv5 algorithm, on the other hand, has faster, more practical, and smaller weight files than its competitors in real-time image processing models^42^. IO lesions are continuous with the surrounding trabecular bone without a capsule^2^ and sometimes do not appear as very prominent opacities. In addition, rapid diagnosis using panoramic radiographs with complex structures and different contrast levels may vary according to the dentist's experience. In this study, it was seen that the YOLOv5 deep learning algorithm could detect IO with high accuracy, even in such lesions and panoramic images. As a result of our intensive literature research, one study detects IO with deep learning. Yesiltepe et al. performed IO detection in the GoogLeNet Inception V2 Faster R-CNN deep learning model using 493 panoramic radiographs. The employed Faster R-CNN structure in this study operates as a two-shot model, engaging in the region proposal first and subsequently performing object detection and classification. It does not possess many of the advantages previously mentioned for single-shot models like YOLOv5. The recall (sensitivity), precision, and F1-Score values of 52 test images were obtained as 0.88, 0.83, and 0.86, respectively. When the results were examined, our study gave higher results in terms of test performance criteria^34^.

The Inception V2 (a faster R-CNN architecture) and YOLO models are employed to identify objects within images or video frames. A study comparing YOLO and faster R-CNN in the detection and classification of breast cancer in mammography images revealed that the modified YOLO v5x demonstrated higher accuracy than YOLO v3 and Faster R-CNN models^61^. The study concluded that YOLO v5 holds promise in distinguishing between benign and malignant breast tumors. Unlike faster R-CNN, the YOLO network can simultaneously perform classification and bounding box regression, resulting in faster processing^62^. The speed of the algorithm, as well as its accuracy, is important in a busy dental workflow. Yesiltepe et al. did not report the inference time; however, the average inference time in the present study was 25.4 ms. YOLO examines the entire bone radiograph in a single pass, enabling it to simultaneously detect and classify bone lesions across the entire radiographic field. YOLO, which was preferred in this study, has important advantages. Firstly, it operates swiftly for detection and classification tasks without the need for a complex pipeline. Secondly, the YOLOv5 variant incorporates a self-adapting anchor, enhancing its capability to detect small objects^63^.

Apart from the jaws, Xiong et al.^64^ differentiated osteoblastic bone metastasis and bone island using the ResNet-18 deep learning model in their study conducted with chest and abdomen CT scans of 728 patients. The accuracy, sensitivity, and specificity were found to be 0.854, 0.731, and 0.987, respectively. Osteoblastic bone metastases might exhibit a resemblance to a bone island, potentially leading to an initial misdiagnosis. This misidentification could result in a delay in the treatment of osteoblastic bone metastases in individuals with tumors. Those two bone lesions are distinguished by features such as thorny radiation, periosteal reaction, soft tissue involvement, and bone destruction. These characteristics are absent in bone islands, and the deep learning algorithm can likely make accurate determinations by learning the absence of these features. As a limitation of our study, different opaque lesions (such as a cemento-osseous dysplasia or an odontoma) were not compared. In such a study design, the deep learning algorithm is expected to learn by extracting the different radiological features (radiodensity, location, expandability, periphery) of lesions that may have radiopaque characteristics and to detect the lesion correctly. IO is nonexpansile and non-capsulated. On the other hand, odontomas may be expansile and show a low-attenuation halo. Both cementoblastoma and cemento-osseous dysplasia are periapical lesions with a sclerotic, well-defined margin and a low-attenuation halo. However, cementoblastoma directly merges with the tooth root, whereas cemento-osseous dysplasia does not fuse to the tooth root^65^. As a result of training deep learning algorithms with such different radiological diagnostic features, it is possible to successfully detect different pathologies on the same image. In another study, Park et al.^66^ employed deep learning models to classify bone tumors (benign, malignant, or no tumor) in the proximal femur based on plain radiographs. The diagnostic accuracy of the model (0.853) was found to be significantly higher than that of the four doctors and the researchers concluded that AI-driven technology has the potential to decrease the likelihood of misdiagnosis among non-specialist doctors in the field of musculoskeletal oncology. Typically, human physicians employ "pattern recognition" to diagnose bone tumors, considering factors such as the tumor's location, shape, size, density, and margin. Features utilized for diagnosing bone tumors in radiographs, such as shape, matrix, density, and transition zone, are deemed appropriate for integration into the deep learning algorithm. Similarly, it appears possible to distinguish features enabling the recognition of IO from other radiopaque lesion features through algorithmic detection.

The first limitation of this study is the small number of data. The IO is a rare lesion^67^ and is not seen in every panoramic radiograph. For this reason, training and testing were done with less data than many deep-learning studies, but high-performance results were obtained. At this stage, the dataset was augmented twofold through data augmentation. Subsequently, the images provided to the YOLOv5 input undergo mosaic data augmentation, inherent to the model's structure. Mosaic images are automatically generated through random scaling, rotation, positioning, and cropping operations on the data, thereby enhancing the model's generalization ability by exposing it to more complex and diverse inputs. By means of these two processes, attempts have been made to overcome data limitations through the utilization of various image manipulation techniques. The second limitation is the absence of central lesions (such as ossifying fibroma, odontoma, etc.) that may require differential diagnosis with IO in the panoramic images included in the study. While the incidence of IO in the Turkish population is 2.7%, the incidence of other radiopaque lesions is lower^57,58^. Therefore, collecting other radiopaque lesions for such a study requires a long time. However, if such a data set can be collected, the deep learning application performed in this study is a good resource for future studies with high success results.

## Conclusıon

In this study, automatic detection and localization of IO on panoramic images with the YOLOv5 deep learning algorithm were successfully performed. For advanced research, this study may provide a perspective for the simultaneous detection and classification of radiolucent and radiopaque lesions with deep learning algorithms. It is expected technology in the future artificial intelligence-based medical image analysis systems will be embedded in hospital PACS systems and brought to doctors and dentists as preliminary information for diagnosis.

## Data Availability

The datasets generated and analyzed during the current study are available from the corresponding author upon reasonable request.

## References

[1] Marques Silva L, Guimaraes AL, Dilascio ML, Castro WH, Gomez RS (2007). A rare complication of idiopathic osteosclerosis. Med. Oral Patol. Oral Cir. Bucal..

[2] Moshfeghi M, Azimi F, Anvari M (2014). Radiologic assessment and frequency of idiopathic osteosclerosis of jawbones: An interpopulation comparison. Acta Radiol..

[3] Sisman Y, Ertas ET, Ertas H, Sekerci AE (2011). The frequency and distribution of idiopathic osteosclerosis of the jaw. Eur. J. Dent..

[4] McDonnell D (1993). Dense bone island: A review of 107 patients. Oral Surg. Oral Med. Oral Pathol..

[5] Greenspan A (1995). Bone island (enostosis): Current concept-a review. Skeletal Radiol..

[6] Gamba TO, Maciel NAP, Rados PV, da Silveira HLD, Arús NA, Flores IL (2021). The imaging role for diagnosis of idiopathic osteosclerosis: A retrospective approach based on records of 33,550 cases. Clin. Oral Investig..

[7] Austin BW, Moule AJ (1984). A comparative study of the prevalence of mandibular osteosclerosis in patients of Asiatic and Caucasian origin. Aust. Dent. J..

[8] Halse A, Molven O (2002). Idiopathic osteosclerosis of the jaws followed through a period of 20–27 years. Int. Endod. J..

[9] Shinde, P. P. & Shah, S. A review of machine learning and deep learning applications. in *2018 Fourth International Conference on Computing Communication Control and Automation (ICCUBEA), IEEE*, 1–6 (2018).

[10] Janiesch C, Zschech P, Heinrich K (2021). Machine learning and deep learning. Electron. Mark..

[11] Park C, Took CC, Seong JK (2018). Machine learning in biomedical engineering. Biomed. Eng. Lett..

[12] Pouyanfar S, Sadiq S, Yan Y (2018). A survey on deep learning: Algorithms, techniques, and applications. ACM Comput. Surv. (CSUR).

[13] Terven, J. & Cordova-Esparza, D. *A Comprehensive Review of YOLO: From YOLOv1 to YOLOv8 and Beyond*. (2023). arXiv:2304.00501.

[14] Jiang P, Ergu D, Liu F, Cai Y, Ma B (2022). A review of yolo algorithm developments. Procedia Comput. Sci..

[15] Mohammad-Rahimi H, Rokhshad R, Bencharit S, Krois J, Schwendicke F (2023). Deep learning: A primer for dentists and dental researchers. J. Dent..

[16] Rabecka, V. D. & Pari, J. B. Assessing the performance of advanced object detection techniques for autonomous cars. in *2023 International Conference on Networking and Communications (ICNWC): IEEE*, 1–7 (2023).

[17] Sánchez S, Campillo J, Martínez-Santos J (2020). Use of deep learning algorithms for real-time detection of vessels in confined spaces using the Tensorflow framework. J. Phys. Conf. Ser..

[18] Teramoto A, Fujita H, Yamamuro O, Tamaki T (2016). Automated detection of pulmonary nodules in PET/CT images: Ensemble false-positive reduction using a convolutional neural network technique. Med. Phys..

[19] Esteva A, Kuprel B, Novoa RA, Ko J, Swetter SM, Blau HM, Thrun S (2017). Dermatologist-level classification of skin cancer with deep neural networks. Nature.

[20] Kallenberg M, Petersen K, Nielsen M, Ng AY, Pengfei D, Igel C, Vachon CM, Holland K, Winkel RR, Karssemeijer N, Lillholm M (2016). Unsupervised deep learning applied to breast density segmentation and mammographic risk scoring. IEEE Trans. Med. Imaging.

[21] Lin PL, Huang PY, Huang PW (2017). Automatic methods for alveolar bone loss degree measurement in periodontitis periapical radiographs. Comput. Methods Programs Biomed..

[22] Corbella S, Srinivas S, Cabitza F (2021). Applications of deep learning in dentistry. Oral Surg. Oral Med. Oral Pathol. Oral Radiol..

[23] De Tobel J, Radesh P, Vandermeulen D, Thevissen PW (2017). An automated technique to stage lower third molar development on panoramic radiographs for age estimation: A pilot study. J. Forens. Odontostomatol..

[24] Casalegno F, Newton T, Daher R, Abdelaziz M, Lodi-Rizzini A, Schürmann F, Krejci I, Markram H (2019). Caries detection with near-infrared transillumination using deep learning. J. Dent. Res..

[25] Chang HJ, Lee SJ, Yong TH, Shin NY, Jang BG, Kim JE, Huh KH, Lee SS, Heo MS, Choi SC, Kim TI, Yi WJ (2020). Deep learning hybrid method to automatically diagnose periodontal bone loss and stage periodontitis. Sci. Rep..

[26] Dasanayaka, C., Dharmasena, B., Bandara, W. R., Dissanayake, M. B. & Jayasinghe, R. In segmentation of mental foramen in dental panoramic tomography using deep learning. in *2019 14th Conference on Industrial and Information Systems (ICIIS)*, 81–84. (2019).

[27] Jaskari J, Sahlsten J, Järnstedt J, Mehtonen H, Karhu K, Sundqvist O, Hietanen A, Varjonen V, Mattila V, Kaski K (2020). Deep learning method for mandibular canal segmentation in dental cone beam computed tomography volumes. Sci. Rep..

[28] Yang H, Jo E, Kim HJ, Cha IH, Jung YS, Nam W, Kim JY, Kim JK, Kim YH, Oh TG, Han SS, Kim H, Kim D (2020). Deep learning for automated detection of cyst and tumors of the jaw in panoramic radiographs. J. Clin. Med..

[29] Yuce F, Öziç MÜ, Tassoker M (2023). Detection of pulpal calcifications on bite-wing radiographs using deep learning. Clin. Oral Investig..

[30] Kuwana R, Ariji Y, Fukuda M, Kise Y, Nozawa M, Kuwada C, Muramatsu C, Katsumata A, Fujita H, Ariji E (2021). Performance of deep learning object detection technology in the detection and diagnosis of maxillary sinus lesions on panoramic radiographs. Dentomaxillofac. Radiol..

[31] Lee JS, Adhikari S, Liu L, Jeong HG, Kim H, Yoon SJ (2019). Osteoporosis detection in panoramic radiographs using a deep convolutional neural network-based computer-assisted diagnosis system: A preliminary study. Dentomaxillofac. Radiol..

[32] Kwon O, Yong TH, Kang SR, Kim JE, Huh KH, Heo MS, Lee SS, Choi SC, Yi WJ (2020). Automatic diagnosis for cysts and tumors of both jaws on panoramic radiographs using a deep convolution neural network. Dentomaxillofac. Radiol..

[33] Aliaga I, Vera V, Vera M, García E, Pedrera M, Pajares G (2020). Automatic computation of mandibular indices in dental panoramic radiographs for early osteoporosis detection. Artif. Intell. Med..

[34] Yesiltepe S, Bayrakdar IS, Orhan K, Çelik Ö, Bilgir E, Aslan AF (2022). A Deep-learning model for idiopathic osteosclerosis detection on panoramic radiographs. Med. Princ. Pract..

[35] Mariani GC, Favaretti F, Lamazza L, De Biase A (2008). Dense bone island of the jaw: A case report. Oral Implantol..

[36] Yonetsu K, Yuasa K, Kanda S (1997). Idiopathic osteosclerosis of the jaws: Panoramic radiographic and computed tomographic findings. Oral Surg. Oral Med. Oral Pathol. Oral Radiol. Endod..

[37] Laroca, R. A robust real-time automatic license plate recognition based on the YOLO detector. in *International Joint Conference on Neural Networks (ijcnn), IEEE*, 1–10 (2018).

[38] Sadykova D, Pernebayeva D, Bagheri M, James A (2019). IN-YOLO: Real-time detection of outdoor high voltage insulators using UAV imaging. IEEE Trans. Power Deliv..

[39] Redmon, J. & Farhadi, A. YOLO9000: Better, faster, stronger. in *Proceedings of the IEEE Conference on Computer Vision and Pattern Recognition*, 7263–7271 (2017).

[40] Redmon, J. & Farhadi, A. *Yolov3: An Incremental Improvement*. (2018). arXiv:1804.02767.

[41] Bochkovskiy, A., Wang, C. Y., Liao, H. Y. M. *Yolov4: Optimal Speed and Accuracy of Object Detection*. (2020). arXiv:2004.10934.

[42] Jocher, G., Nishimura, K., Mineeva, T., Vilariño, R. *Yolov5. Code Repository*. (2020). https://github.com/ultralytics/yolov5.

[43] Katsamenis, I. *et al*. TraCon: A novel dataset for real-time traffic cones detection using deep learning. in *Novel & Intelligent Digital Systems Conferences*, 382–391 (Springer, 2023).

[44] Lv H, Yan H, Liu K, Zhou Z, Jing J (2022). Yolov5-ac: Attention mechanism-based lightweight yolov5 for track pedestrian detection. Sensors.

[45] Xu R, Lin H, Lu K, Cao L, Liu Y (2021). A forest fire detection system based on ensemble learning. Forests.

[46] Wang, C. Y. *et al*. CSPNet: A new backbone that can enhance learning capability of CNN. in *Proceedings of the IEEE/CVF Conference on Computer Vision and Pattern Recognition Workshops*, 390–391 (2020)

[47] He K, Zhang X, Ren S, Sun J (2015). Spatial pyramid pooling in deep convolutional networks for visual recognition. IEEE Trans. Pattern Anal. Mach. Intell..

[48] Wang, W. *et al*. Efficient and accurate arbitrary-shaped text detection with pixel aggregation network. in *Proceedings of the IEEE/CVF International Conference on Computer Vision*, 8440–8449 (2019).

[49] Lin, T. Y. *et al*. Feature pyramid networks for object detection. in *Proceedings of the IEEE Conference on Computer Vision and Pattern Recognition*, 2117–2125 (2017).

[50] Silva, B., Pinheiro, L., Oliveira, L. & Pithon, M. A study on tooth segmentation and numbering using end-to-end deep neural networks. in *33rd SIBGRAPI Conference on Graphics, Patterns and Images (SIBGRAPI): IEEE*, 164–171 (2020).

[51] Tuzoff DV, Tuzova LN, Bornstein MM, Krasnov AS, Kharchenko MA, Nikolenko SI (2019). Tooth detection and numbering in panoramic radiographs using convolutional neural networks. Dentomaxillofac. Radiol..

[52] Celik ME (2022). Deep learning based detection tool for impacted mandibular third molar teeth. Diagnostics.

[53] Başaran M, Çelik Ö, Bayrakdar IS, Bilgir E, Orhan K, Odabaş A (2021). Diagnostic charting of panoramic radiography using deep-learning artificial intelligence system. Oral Radiol..

[54] Lian L, Zhu T, Zhu F, Zhu H (2021). Deep learning for caries detection and classification. Diagnostics.

[55] Demir A, Pekiner FN (2019). Idiopathic osteosclerosis of the jaws in turkish subpopulation: Cone-beam computed tomography findings. Clin. Exper. Health Sci..

[56] Bsoul SA (2004). Idiopathic osteosclerosis (enostosis, dense bone islands, focal periapical osteopetrosis). Quintessence Int..

[57] Miloglu O, Yalcin E, Buyukkurt MC, Acemoglu H (2009). The frequency and characteristics of idiopathic osteosclerosis and condensing osteitis lesions in a Turkish patient population. Med. Oral. Patol Oral. Cir. Bucal..

[58] Dedeoğlu N, Arıkan B (2021). Evaluation of radiopaque lesions of the jaw bones on digital panoramic radiography in a turkish subpopulation: A retrospective study. Atatürk Üniv. Hekimliği Fakültesi Dergisi.

[59] Yang H, Jo E, Kim HJ, Cha I-h, Jung Y-S, Nam W (2020). Deep learning for automated detection of cyst and tumors of the jaw in panoramic radiographs. J. Clin. Med..

[60] Orhan K, Bayrakdar I, Ezhov M, Kravtsov A, Özyürek T (2020). Evaluation of artificial intelligence for detecting periapical pathosis on cone-beam computed tomography scans. Int. Endod. J..

[61] Mohiyuddin A (2022). Breast tumor detection and classification in mammogram images using modified YOLOv5 network. Comput. Math. Methods Med..

[62] Du J (2018). Understanding of object detection based on CNN family and YOLO. J. Phys. Conf. Ser..

[63] Li J (2023). Primary bone tumor detection and classification in full-field bone radiographs via YOLO deep learning model. Eur. Radiol..

[64] Xiong Y (2023). Deep learning-based diagnosis of osteoblastic bone metastases and bone islands in computed tomograph images: A multicenter diagnostic study. Eur. Radiol..

[65] Curé JK, Vattoth S, Shah R (2012). Radiopaque jaw lesions: An approach to the differential diagnosis. Radiographics.

[66] Park JW (2022). Artificial intelligence-based classification of bone tumors in the proximal femur on plain radiographs: System development and validation. PLoS ONE.

[67] Alkurt MT, Sadık E, Peker İ (2014). Prevalence and distribution of idiopathic osteosclerosis on patients attending a dental school. J. Istanbul Univ. Fac. Dent..

